# DNMT3a‐mediated methylation of PPARγ promote intervertebral disc degeneration by regulating the NF‐κB pathway

**DOI:** 10.1111/jcmm.18048

**Published:** 2023-11-20

**Authors:** Peng Cheng, Hang‐Zhi Wei, Hai‐Wei Chen, Zhi‐Qiang Wang, Peng Mao, Hai‐Hong Zhang

**Affiliations:** ^1^ Department of Emergency Medicine Lanzhou University Second Hospital Lanzhou Gansu PR China; ^2^ Department of Orthopedics Lanzhou University Second Hospital Lanzhou Gansu Province PR China; ^3^ Department of Department of General Surgery Lanzhou University Second Hospital Lanzhou Gansu PR China; ^4^ The Second Clinical Medical College Lanzhou University Lanzhou Gansu PR China

**Keywords:** DNMT3a, intervertebral disc degeneration, low back pain, PPARγ

## Abstract

Intervertebral disc degeneration (IVDD) is a common chronic musculoskeletal disease that causes chronic low back pain and imposes an immense financial strain on patients. The pathological mechanisms underlying IVDD have not been fully elucidated. The development of IVDD is closely associated with abnormal epigenetic changes, suggesting that IVDD progression may be controlled by epigenetic mechanisms. Consequently, this study aimed to investigate the role of epigenetic regulation, including DNA methyltransferase 3a (DNMT3a)‐mediated methylation and peroxisome proliferator‐activated receptor γ (PPARγ) inhibition, in IVDD development. The expression of DNMT3a and PPARγ in early and late IVDD of nucleus pulposus (NP) tissues was detected using immunohistochemistry and western blotting analyses. Cellularly, DNMT3a inhibition significantly inhibited IL‐1β‐induced apoptosis and extracellular matrix (ECM) degradation in rat NP cells. Pretreatment with T0070907, a specific inhibitor of PPARγ, significantly reversed the anti‐apoptotic and ECM degradation effects of DNMT3a inhibition. Mechanistically, DNMT3a modified PPARγ promoter hypermethylation to activate the nuclear factor‐κB (NF‐κB) pathway. DNMT3a inhibition alleviated IVDD progression. Conclusively, the results of this study show that DNMT3a activates the NF‐κB pathway by modifying PPARγ promoter hypermethylation to promote apoptosis and ECM degradation. Therefore, we believe that the ability of DNMT3a to mediate the PPARγ/NF‐κB axis may provide new ideas for the potential pathogenesis of IVDD and may become an attractive target for the treatment of IVDD.

## 1 INTRODUCTION

Intervertebral disc degeneration (IVDD) is a prevalent and chronic musculoskeletal disorder that causes chronic low back pain (LBP).^1^, ^2^ In the lifetime of humans, approximately 80% of people worldwide are affected by LBP, substantially reducing the quality of life of patients, increasing the financial burden and elevating medical resources utilization.^3^, ^4^ Presently, the treatment of IVDD is primarily conservative and surgical, making it difficult to fundamentally delay or reverse its occurrence and progression. Additionally, IVDD treatment is a great challenge for doctors. Factors such as inflammatory response, oxidative stress, autophagy, abnormal mechanical stress, nutritional deficiency and genetics can accelerate IVDD development.^5^, ^6^, ^7^ However, the pathological mechanism of IVDD has not been fully elucidated, and further research is urgently needed to reveal its exact pathological mechanism. Recent studies have shown that IVDD development is closely associated with abnormal epigenetic changes,^8^, ^9^ suggesting that IVDD progression may be controlled by epigenetic mechanisms.

Epigenetic modifications regulate gene expression by influencing transcription or inhibiting translation without involving alterations in DNA sequences.^10^ It generally includes DNA methylation, histone modification, adenosine triphosphate‐dependent chromatin remodelling, and non‐coding RNA interference, of which DNA methylation is one of the most widely studied epigenetic regulatory mechanisms.^11^ DNA methylation is regulated by DNA methyltransferase 3a (DNMT3a) and DNA methyltransferase 3b (DNMT3b), which are maintained by DNA methyltransferase 1 (DNMT1).^12^ DNA is usually methylated at the C‐phosphate‐G (CpG) island cytosine base 5 in gene promoters or enhancers, thereby silencing the transcriptional activity of the target genes.^13^ Recent genomic studies have shown significant differences in the DNA methylation profiles between mildly and severely degenerative human intervertebral discs (IVD), suggesting that DNA methylation is involved in the human IVDD process.^14^ Recent studies have shown that DNMT3b m6A hypomethylation regulated by ALKBH5 promotes disc degradation through E4f1 defects, suggesting that DNA methylation plays an important role in the pathological process of IVDD.^15^ However, key genes involved in regulating DNMT3a methylation that may control the development of IVDD and have therapeutic potential remain unclear.

Peroxisome proliferator activates receptor‐γ (PPARγ), a member of the nuclear hormone receptor family, is involved in various pathophysiological processes, such as regulating lipid and sugar metabolism, inhibiting inflammation, inducing tumour cell differentiation and apoptosis, and anti‐atherosclerosis.^16^ There are three forms of PPARγ; particularly, the expression of PPARγ2 and PPARγ3 is present in adipose tissue and macrophages, while PPARγ1 is widely expressed in many tissues and has high expression in articular cartilage.^17^ Studies have found that mice lacking PPARγ show accelerated development of osteoarthritis compared with wild‐type mice,^18^, ^19^ indicating that PPARγ is essential for maintaining chondrocyte homeostasis. Recent studies have shown that PPARγ activator can inhibit the degradation of the extracellular matrix (ECM) of the nucleus pulposus (NP), thereby delaying the progression of IVDD,^20^, ^21^ suggesting that PPARγ may be a potential therapeutic target for IVDD. However, the specific molecular mechanism that mediates PPARγ remains unclear. NF‐κB pathway plays a crucial role in the pathological processes of IVDD.^22^ Inhibition of the NF‐κB pathway can reduce the ECM catabolism, inflammation, apoptosis, senescence and pyroptosis of NP cells.^23^, ^24^ However, whether PPARγ can participate in IVDD development by regulating the NF‐κB pathway activity remains unclear. Consequently, this study aimed to explore whether PPARγ can alleviate IVDD through the NF‐κB pathway.

In this study, we found that DNMT3a‐mediated PPARγ promoter hypermethylation plays a crucial role in IVDD development. Furthermore, we observed that enhancing PPARγ expression through promoter demethylation inhibited the NF‐κB signalling pathway and slowed IVDD development. Therefore, we inferred that increased PPARγ activity could delay IVDD development and that PPARγ may be a potential target for the treatment of IVDD.

## 2 METHODS

### 2.1 Human NP tissue collection and classification

The magnetic resonance imaging (MRI) data of human NP tissues were classified according to the Pfirrmann grading method.^25^ Discs categorized as Pfirrmann Grade I or II was considered as early IVDD group, and Pfirrmann Grade III, IV, or V was classified as late IVDD group. Early NP tissue was collected from patients diagnosed with idiopathic scoliosis and lumbar fractures (5 females and 3 males; age 19.4 ± 4.88; Grade I [*n* = 3] and Grade II [*n* = 5]), and late NP tissue was collected from patients diagnosed with lumbar disc herniation or lumbar spondylolisthesis undergoing disc fusion surgery (14 females and 10 males; age 43 ± 6.42 years; Grade III [*n* = 8], Grade IV [*n* = 8] and Grade V [*n* = 8]). Pfirrmann Grades I–V tissue samples were randomly selected three for immunohistochemistry (IHC) analysis. The remaining NP tissue samples were frozen in liquid nitrogen and stored for western blotting analysis. All patients provided written informed consent, and all protocols were approved by the Ethics Committee of the Second Hospital of Lanzhou University (2023A‐588).

### 2.2 Culture of rat nucleus pulposus cells

The extraction method for Sprague–Dawley rat NP cells was based on our previous research.^26^ Extracted NP cells were cultured in flasks containing Dulbecco's Modified Eagle Medium (DMEM)/F12 medium containing 10% fetal bovine serum (FBS) in an incubator at 37°C, 5% CO_2_. When the cells reached approximately 90% confluency, NP cells were dissociated using 0.25% trypsin and subcultured. Third‐generation NP cells were used for the subsequent experiments in this study.

### 2.3 Lentiviral transfection

DNMT3a short hairpin RNA (shRNA) and scrambled shRNA were purchased from Gemma (Shanghai, China). Transfection with DNMT3a shRNA inhibited DNMT3a expression Lentiviruses containing DNMT3a shRNA or negative control lentivirus (shRNA‐NC) were added to NP cells at a multiplicity of infection (MOI) of 100. NP cells were cultured in a 37°C, 5% CO_2_ incubator. After 24 h, the medium was changed, and the cells were cultured for 3 days and transferred for subsequent experiments. Transfection efficiency was determined using western blotting and quantitative reverse transcriptase polymerase chain reaction (qRT‐PCR). The sequences were as follows:

Dnmt3a‐Rat‐2182‐1:5′‐TCGCTCCGCTGAAGGAATATT‐3′.

Dnmt3a‐Rat‐764‐2:5′‐GAATCCTTACAAGGAAGTTTA‐3′.

Dnmt3a‐Rat‐1292‐3:5′‐CCAGATGTTCTTCGCCAATAA‐3′.

### 2.4 Western blotting assay of protein expressions

Human and rat NP tissues were lysed in a lysis buffer containing protease inhibitors for 30 min. The cell lysate was centrifuged at 12,000 rpm, 4°C for 15 min. Protein detection in the supernatant was performed using a Bradford Protein Assay Kit. Equal‐sized protein samples were separated using 10% sodium dodecyl sulfate (SDS)‐polyacrylamide gel electrophoresis and transferred to a polyvinylidene fluoride membrane (Millipore, Billerica, MA, USA). The membrane was blocked with 5% bovine serum albumin for 2 h at room temperature, then incubated overnight at 4°C with primary antibodies against the following proteins: DNMT3a (anti‐rabbit, 1:1000), PPARγ (anti‐rabbit, 1:1000), Caspase‐3 (anti‐rabbit, 1:1000), Bcl‐2 (anti‐rabbit, 1:1000), Bax (anti‐rabbit, 1:1000), Collagen II (anti‐rabbit, 1:1000), Aggrecan (anti‐rabbit, 1:1000), Adamts‐4 (anti‐rabbit, 1:1000), MMP‐3 (anti‐rabbit, 1:1000), p‐P65 (anti‐rabbit, 1:1000), P65 (anti‐rabbit, 1:1000), p‐IKBα (anti‐rabbit, 1:1000), IKBα (anti‐rabbit, 1:1000) and β‐actin (anti‐mouse, 1:1000). Subsequently, the membrane was washed thrice for 10 min each and incubated with horseradish peroxidase‐conjugated goat anti‐rabbit antibody (1:5000; Beyotime) or horseradish peroxidase‐conjugated goat anti‐mouse antibody (1:5000; Beyotime) at room temperature for 2 h. ImageJ software (https://imagej.nih.gov/ij/) was used to analyse the western blot data.

### 2.5 qRT‐PCR

Total RNA was extracted from rat NP cells using AG RNAex Pro Reagent (Accurate Biotechnology, Ltd., China), and the absorbance ratios at 260/280 nm and 260/230 nm were determined using a UV–Vis spectrophotometer (Thermo, USA) to assess the purity and concentration of total RNA in each sample. Reverse transcription of mRNA into cDNA was conducted using Evo M‐MLV RT Kit. Subsequently, qRT‐PCR was performed using the SYBR Green Premix Pro Taq HS qPCR Kit (Accurate Biotechnology Ltd., China). The primers used were as follows: Rat DNMT3a, forward 5’‐AAGGACCCTGCGGTGATCT‐3,’ reverse 5′‐TTGGGTAATAGCTCTGAGGCG‐3′; GAPDH, forward 5′‐GGAAGCTTGTCATCAATGGAAATC‐3′; reverse 5′‐TGATGACCCTTTTGGCTCCC‐3′. Three replicate wells were set up per reaction to quantify the gene expression of interest using the 2^−ΔΔCt^ method.

### 2.6 Methylation‐specific PCR (MSP)

The CpG island of the rat PPARγ promoter was analysed using MetPrimer software (http://www.urogene.org/methprimer/), and MSP primers were designed. Genomic DNA was extracted from NP cells according to the instructions provided with the EasyPure® Genomic DNA Kit (Beijing, China). DNA was modified with bisulfite using a DNA Bisulfite Conversion Kit (TIANGEN, Beijing, China). PCR products were analysed on agarose gels for density using ImageJ software. Methylated and unmethylated PPARγ primer sequences were as follows: Methylated primers: forward 5′‐CGTGGGATTTTTGTGGC‐3′, Reverse 5′‐CACGAAACCCGGTAACT‐3′; Unmethylated primers: Forward 5′‐GAATGTGGGATTTTTGTGTT‐3′, Reverse 5′‐CCCACAAAACCCACATAACTC‐3.

### 2.7 Immunofluorescence staining

Rat NP cells were transferred to flat‐bottomed 24‐well plates at a specified density (5 × 10^3^ cells/well). After treatment, cells were fixed with 4% paraformaldehyde, followed by permeability with 0.5% tritonX‐100 for 30 min, blocked with 5% BSA, and incubated overnight at 4°C with corresponding antibodies. After washing, cells were incubated with DyLight 649 or DyLight 488 (Abbkine) anti‐rabbit secondary antibody, which was diluted at 1:300, incubated at 37°C for 1 h, and then photographed using an inverted fluorescence microscope (Olympus).

### 2.8 Terminal deoxynucleotidyl transferase‐mediated dUTP nick end labeling (TUNEL) staining

DNA damage level was detected using TUNEL staining. NP cells were seeded into 24‐well plates, fixed with fresh 4% paraformaldehyde for 30 min, and incubated with 3% H_2_O_2_ and 0.5% Triton X‐100 for 30 min. Finally, according to the manufacturer's instructions, the cells and the TUNEL reaction mixture were incubated at 37°C for 1 h and counterstained with 4′,6‐diamidino‐2‐phenylindole (DAPI) for 15 min in the dark. After washing the cells thrice with PBS at room temperature in the dark, images of the cells were obtained using a fluorescence microscope (Olympus, Tokyo, Japan). Apoptotic cells showed dense fluorescent particles in the nucleus.

### 2.9 Cell viability assay

Cell Counting Kit‐8 (CCK‐8; Yeasen Biotechnology) was used to measure NP cell viability. NP cells were seeded in 96‐well plates containing 100 μL of whole medium (containing 10% FBS) and incubated for 24 h in a 37°C 5% CO_2_ incubator. PPARγ inhibitor (T0070907) (Medchem Express, Shanghai, China) was added to 5, 10, 15, 25, 50 and 100 μM complete medium culture for 24 h; on the next day, 10 μL of CCK‐8 solution per well was added and incubated at 37°C for 2 h. Finally, the 450 nm optical density (OD450) was measured using a microplate analyser (Bio‐Rad, Hercules, CA, USA). Similarly, another batch of cells was used to detect cell viability of the PPARγ activator (GW1929) (Medchem Express, shanghai, China) and NF‐κB pathway activator (diprovocim) (Medchem Express, shanghai, China). The percentage cell viability was calculated as $\left(1-\left[=\text{mean}\;\mathrm{OD}\;\text{in the drug group}/\text{mean}\;\mathrm{OD}\;\text{in the control group}\right]\right)\times 100\%$.

### 2.10 Animal experiments

Adult male Sprague–Dawley rats (200–250 g) were purchased from Lanzhou Veterinary Research Institute in Gansu Province. The rats were placed at an indoor temperature of 23 ± 2°C, were alternately reared under light and dark conditions for 12 h, and were fed regular supplementation with feed and purified water. A classic rat fibre ring‐needled IVDD model was used as previously described.^27^ All experimental rats were anaesthetised using pentobarbital sodium (40 mg/kg, intraperitoneal injection). They were pierced with a 21G needle at Co6‐7, CO7‐8, and CO8‐9, rotated 180°, and held for 30 s. Subsequently, we administered a 2 μL PBS injection into the NP of Co6‐7, 2 μL of shRNA‐NC into the NP of Co7‐8, and 2 μL of shRNA‐DNMT3a into the NP of Co8‐9. The rats were returned to their cages and fed continuously.

### 2.11 X‐ray and MRI scan

All rats' tails underwent X‐ray and MRI 8 weeks after surgery. The rats remained in a horizontal supine position with the tail extending straight on the mammography device (GE). X‐ray irradiation was performed at a distance of 66 cm between the collimator and film, penetration voltage of 35 kV and exposure intensity of 63 mA. The sagittal T2‐weighted MRI image was acquired using a 3.0 T clinical magnet with a fast‐spin echo sequence with a time‐to‐repetition of 5400 ms, a time‐to‐echo of 920 ms, a 320 (h) × 256 (v) matrix, a field‐of‐view of 260°, and four excitations. The section thickness was 2 mm, and the gap was 0 mm. After the X‐ray and MRI examinations were completed, the rats were euthanized, and images were acquired and analysed.

### 2.12 Haematoxylinand eosin (HE) staining, toluidine blue staining and safranine O staining

Fresh 4% paraformaldehyde was used to fix human NP tissue and rat NP tissue, which was embedded in paraffin and then sectioned into 5 μm sections for use. HE, toluidine blue and Safranine O stainings were performed according to the manufacturer's instructions. After staining, the seal was fixed with gel and photographed using an EVOS microscope (EVOS‐FL Cell imaging System, Thermo Fisher Scientific).

### 2.13 IHC staining

Fresh 4% paraformaldehyde was used to fix human NP tissue and rat NP tissue, which was embedded in paraffin and then sectioned into 5 μm sections for use. Endogenous peroxidase activity was blocked with 3% H_2_O_2_ for 15 min and then incubated with 5% bovine serum albumin (BSA) and 1% Tween‐20 in phosphate‐buffered saline (PBS) for 30 min to block non‐specific antigens. The tissue sections were incubated overnight with anti‐PPARγ, DNMT3a, Collagen II, MMP‐3 and Caspase‐3 antibodies at 4°C. Subsequently, tissue sections were incubated with the corresponding enzyme‐labelled secondary antibody (Proteintech, 1:5000) and counterstained with haematoxylin. Photographic records were generated using a panoramic histiocyte scanner and analysed using the ImageJ software.

### 2.14 Statistical analysis

Data were analysed using GraphPad Prism 9 software, and all experiments were repeated at least thrice. We used t‐tests and one‐way or two‐way analysis of variance (anova) to compare data from different groups. The Kruskal–Wallis test was used to analyse differences between groups. The values of *p* < 0.05 (*p < 0.05, **p < 0.01, ***p < 0.001) were considered statistically significant.

## 3 RESULTS

### 3.1 Expression of DNMT3a and PPARγ in human NP tissues

**FIGURE 1:**
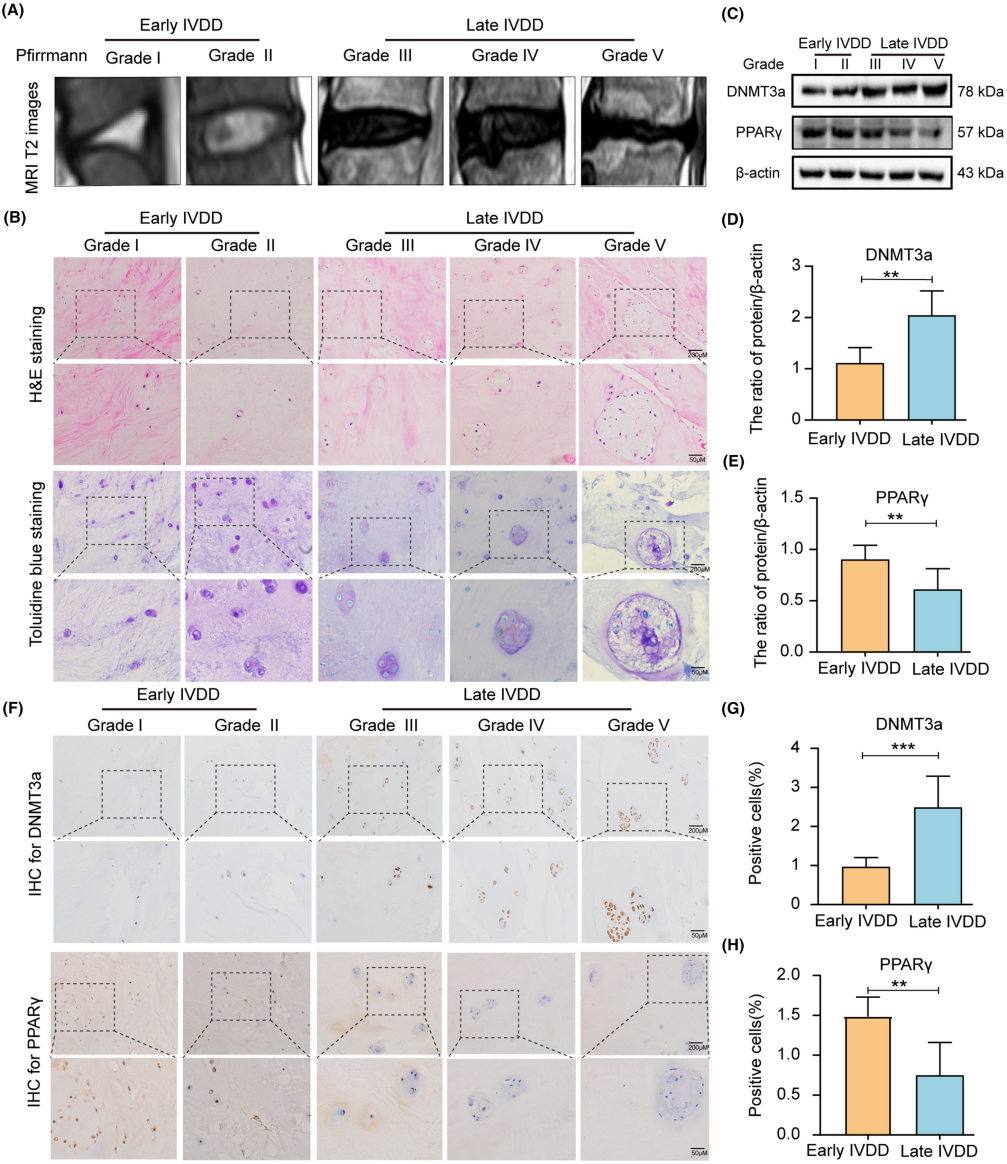
Expression of DNMT3a and PPARγ in human NP tissues; (A) MRI T2 images of human NP tissue according to the Pfirrmann grades; (B) Haematoxylin and eosin staining (HE) and toluidine blue staining of early and late human NP tissues; (C) Western blotting analysis of DNMT3a and PPARγ in early and late human NP tissues; (D, E) Relative quantitative protein levels of DNMT3a and PPARγ; (F) Expression of DNMT3a and PPARγ by immunochemical staining in early and late human NP tissues; (G, H) Quantitative levels of immunohistochemical staining of DNMT3a and PPARγ in early and late human NP tissues; Three biological replicates were contained in each experiment. All data are expressed as mean ± standard deviation (SD); **p < 0.01, ***p < 0.01.

Early and late human NP tissue samples were collected, and the degree of NP tissue degeneration was assessed based on the patient's T2 MRI images (Figure 1A). Haematoxylin and eosin (HE) and toluidine blue staining showed a change in the characteristics of NP tissues during the progression of IVDD. In the early IVDD group, the collagen fibres in NP tissues were neatly arranged, and the ECM staining of NP cells was uniform. In the late IVDD group, the collagen fibres in the NP tissue were highly disordered, and the NP cells were clustered in large quantities, showing large vacuolar cells (Figure 1B). Additionally, western blotting analysis showed increased DNMT3a expression and decreased PPARγ expression in late IVDD group, compared with early IVDD group (Figure 1C–E). Further, the expression levels of DNMT3a and PPARγ were measured in human NP tissues by immunohistochemical analysis. Compared to early NP tissues, DNMT3a expression in late NP tissues was increased and PPARγ expression was decreased (Figure 1F–H). These results suggest that DNMT3a level increase, and PPARγ level gradually decrease during the IVDD progression.

### 3.2 IL‐1β treatment causes increased expression of DNMT3a and decreased expression of PPARγ in rat NP cells

**FIGURE 2:**
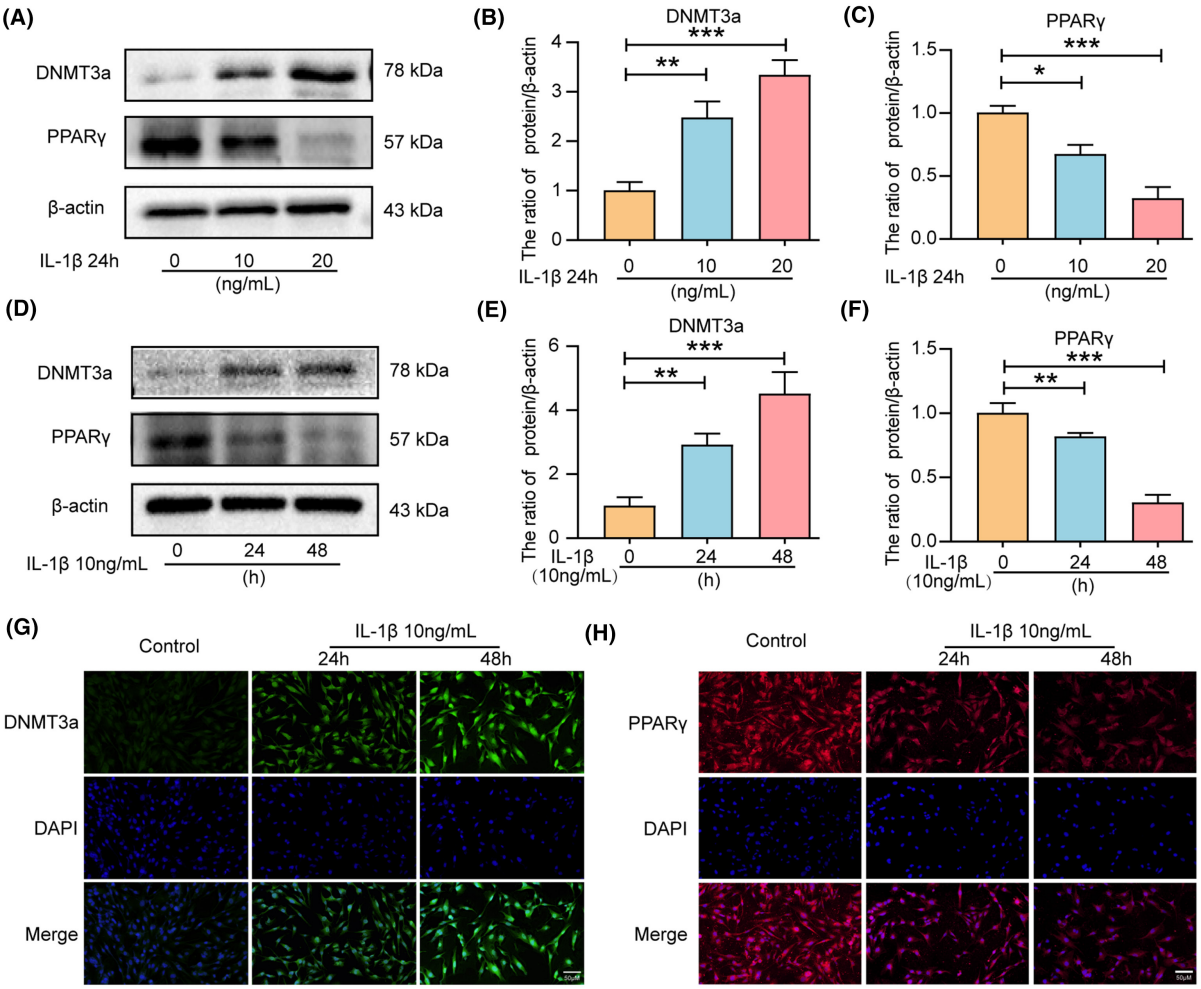
IL‐1β treatment can cause increased DNMT3a expression and decreased PPARγ expression in NP cells. (A–F) Western blotting was used to measure the expression of DNMT3a and PPARγ and the relative quantitative protein levels of DNMT3a and PPARγ in NP cells treated with different IL‐1β concentrations and times; (G, H) IL‐1β treated DNMT3a and PPARγ representative immunofluorescence staining in NP cells at different times; Three biological replicates were contained in each experiment. All data are expressed as mean ± standard deviation (SD); *p < 0.05, **p < 0.01, ***p < 0.01.

Inflammation is considered an important contributing factor to IVDD progression.^28^ IL‐1β can significantly increase the expression of apoptosis‐associated proteins (Caspase‐3, Bax, and Bcl‐2) in IVD cells in vitro, promoting apoptosis and ECM degradation in NP cells.^29^, ^30^ Therefore, we evaluated the changes in PPARγ and DNMT3a in IL‐1β‐treated NP cells. Western blot analysis revealed that IL‐1β treatment increased the levels of DNMT3a expression while reducing PPARγ levels in NP cells in a dose‐dependent manner (Figure 2A–C) and time‐dependent manner (Figure 2D–F). Therefore, the subsequent experiment selected IL‐1β concentration of 10 ng/mL for 24 h. Additionally, immunofluorescence analysis confirmed that the DNMT3a expression increased and the PPARγ expression decreased in IL‐1β‐treated NP cells (Figure 2G,H). These results are consistent with the findings in human tissue studies that DNMT3a was up‐regulated, and PPARγ was down‐regulated during IL‐1β‐induced NP cell degeneration.

### 3.3 DNMT3a inhibition attenuates IL‐1β‐induced apoptosis and ECM degradation

**FIGURE 3:**
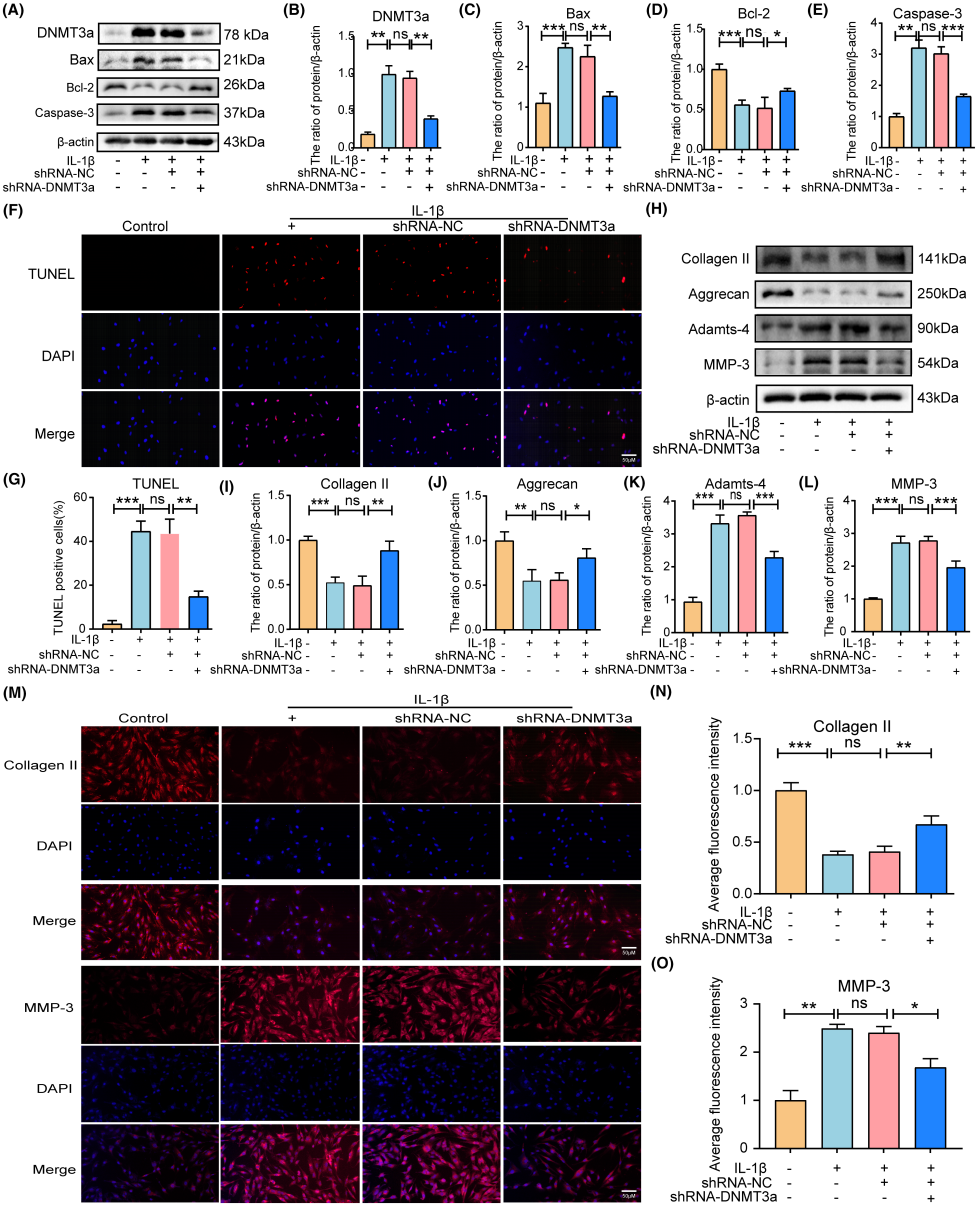
DNMT3a inhibition attenuates IL‐1β‐induced apoptosis and ECM degradation. (A–E) Western blotting and quantitative analyses show western blot levels of DNMT3a, Bax, Bcl‐2, and Caspase‐3 in rat NP cells in different groups; (F, G) TUNEL staining and apoptotic cell ratio quantification. (H–L) Western blotting and quantitative analyses show levels of Collagen II, Aggrecan, Adamts‐4, and MMP‐3 in different groups; (M–O) immunofluorescence and quantitative analysis showed changes in Collagen II and MMP‐3. Three biological replicates were contained in each experiment. All data are expressed as mean ± standard deviation (SD); *p < 0.05, **p < 0.01, ***p < 0.01, ns *p* > 0.05.

To investigate whether silencing DNMT3a inhibits the apoptosis of IL‐1β‐induced rat NP cells in vitro, we infected lentiviruses with scrambled shRNA (shRNA‐NC) and DNMT3a shRNA (shRNA‐DNMT3a) and assessed the silencing efficiency using qRT‐PCR and western blotting (Supporting Information S1). Western blotting results showed that DNMT3a inhibition significantly reversed the IL‐1β‐induced increase in DNMT3a expression (Figure 3A,B). Apoptosis is often accompanied by the upregulation of Caspase‐3 and Bax expression and the downregulation of Bcl‐2. As shown in Figure 3A,C–E, IL‐1β promoted the expression of Bax and Caspase‐3 pro‐apoptotic proteins and reduced the expression level of anti‐apoptotic protein Bcl‐2 compared to the control group (*p* < 0.05). Additionally, the shRNA‐DNMT3a group, compared with the IL‐1β and shRNA‐NC groups, experienced significantly reduced pro‐apoptotic proteins (Bax and Caspase‐3) expression and increased anti‐apoptotic proteins (Bcl‐2) expression, indicating that the silencing of DNMT3a can inhibit the apoptosis of IL‐1β‐induced NP cells (Figure 3A,C–E). Moreover, TUNEL staining showed that shRNA‐DNMT3a significantly reduced the IL‐1β‐induced NP cells apoptosis ratio (Figure 3F,G).

Furthermore, we evaluated whether inhibition of DNMT3a inhibits IL‐1β‐induced ECM degradation in rat NP cells. The balance between ECM synthesis and degradation contributes to the structural stability of intervertebral discs, which is a key indicator of NP cell function. We examined the expression of matrix decomposition enzymes (MMP‐3 and Adamts‐4), collagen II, and aggrecan in NP cells to assess the balance between ECM synthesis and degradation in NP cells. As shown in Figure 3H–L, IL‐1β promoted MMP‐3 and Adamts‐4 expression levels and decreased collagen II and aggrecan expression levels compared with the control group (*p* < 0.05). Additionally, shRNA‐DNMT3a increased collagen II and aggrecan expression levels and decreased MMP‐3 and Adamts‐4 expression levels compared to the IL‐1β and shRNA‐NC groups (Figure 3H–L). Additionally, immunofluorescence staining showed that shRNA‐DNMT3a significantly increased collagen II expression levels and decreased MMP‐3 expression levels (Figure 3M–O). These results suggest that DNMT3a silencing inhibits NP apoptosis and ECM degradation.

### 3.4 DNMT3a inhibits PPARγ expression by modifying PPARγ promoter methylation

**FIGURE 4:**
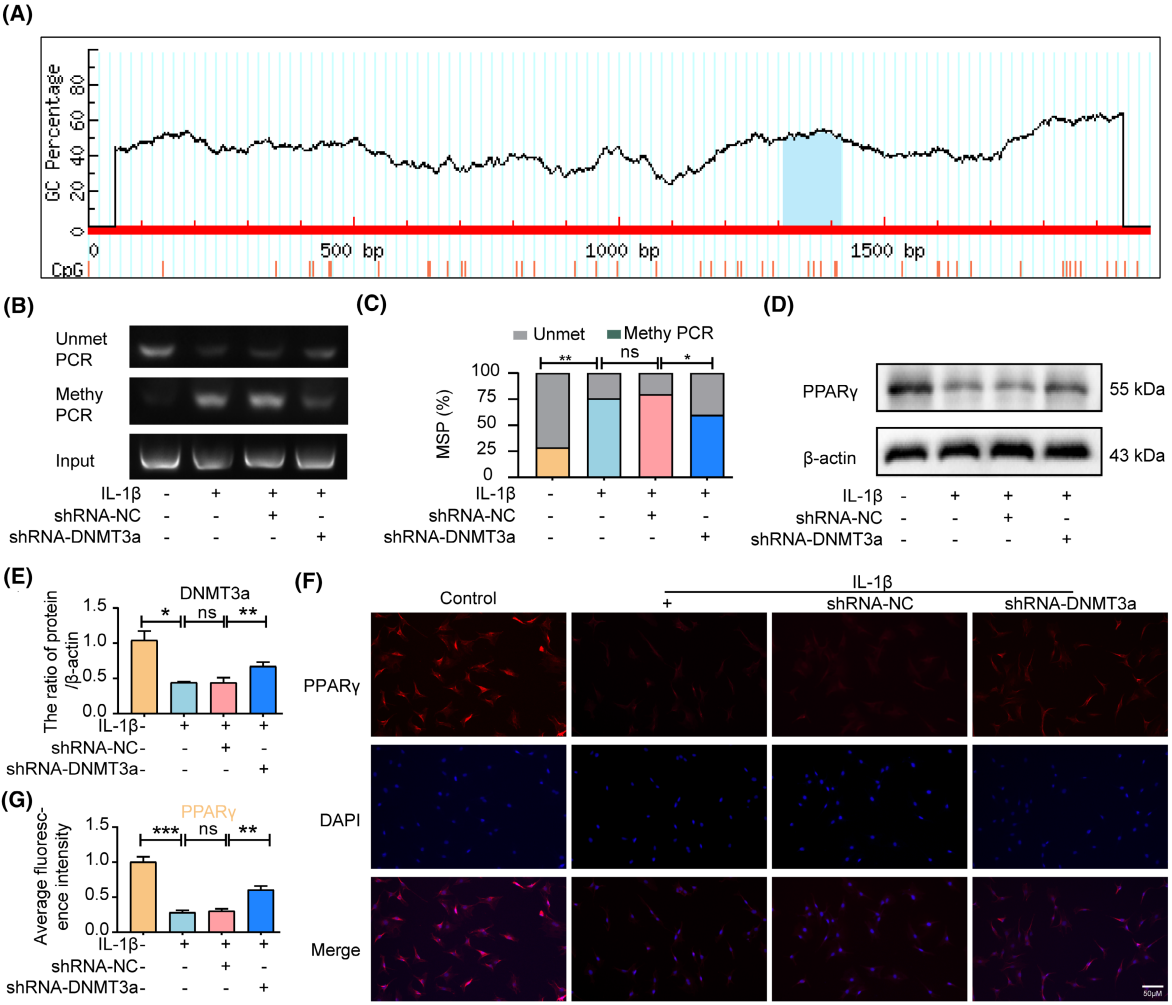
DNMT3a inhibits PPARγ expression by modifying PPARγ promoter methylation. (A) The island of C‐phosphate‐G (CpG) in the PPARγ promoter region was found on the UCSC website; (B, C) methylation and quantification of PPARγ promoters in NP cells by MSP detection 24 h after transfection of shRNA‐DNMT3a; (D, E) Western blotting and quantification analyses show levels of PPARγ after IL‐1β and shRNA‐DNMT3a treatment; (F, G) immunofluorescence and quantitative analyses show changes in PPARγ. Three biological replicates were contained in each experiment. All data are expressed as mean ± standard deviation (SD); *p < 0.05, **p < 0.01, ***p < 0.01, ns *p* > 0.05.

The UCSC Genomic Explorer database (http://genome.ucsc.edu) was used to predict the PPARγ promoter CpG island region. As shown in Figure 4A, the PPARγ promoter in rats has a typical CpG island located in the 1312/1418 region. To further clarify the epigenetic mechanism of PPARγ inhibition due to abnormal DNA methylation, we used shRNA‐NC or shRNA‐DNMT3a to transfect NP cells and detected the DNA methylation status of PPARγ promoters in rat NP cells (1312/1418 sites) using MSP analysis. The MSP results showed that the methylation degree of the PPARγ promoter was significantly increased after IL‐1β treatment in rat NP, whereas the activity of DNMT3a was inhibited after shRNA‐DNMT3a treatment compared with the IL‐1β and shRNA‐NC groups, resulting in a significant decrease in the degree of methylation of the PPARγ promoter (Figure 4B,C). Moreover, western blotting results showed that the shRNA‐DNMT3a group experienced significantly increased PPARγ expression levels compared with the IL‐1β and shRNA‐NC groups (Figure 4D,E). Furthermore, immunofluorescence and quantitative analyses showed that shRNA‐DNMT3a significantly increased PPARγ expression levels compared to the IL‐1β and shRNA‐NC groups (Figure 4F,G). The above results show that DNMT3a inhibits the expression of PPARγ by modifying PPARγ promoter methylation.

### 3.5 DNMT3a inhibits PPARγ to promote apoptosis and ECM degradation

**FIGURE 5:**
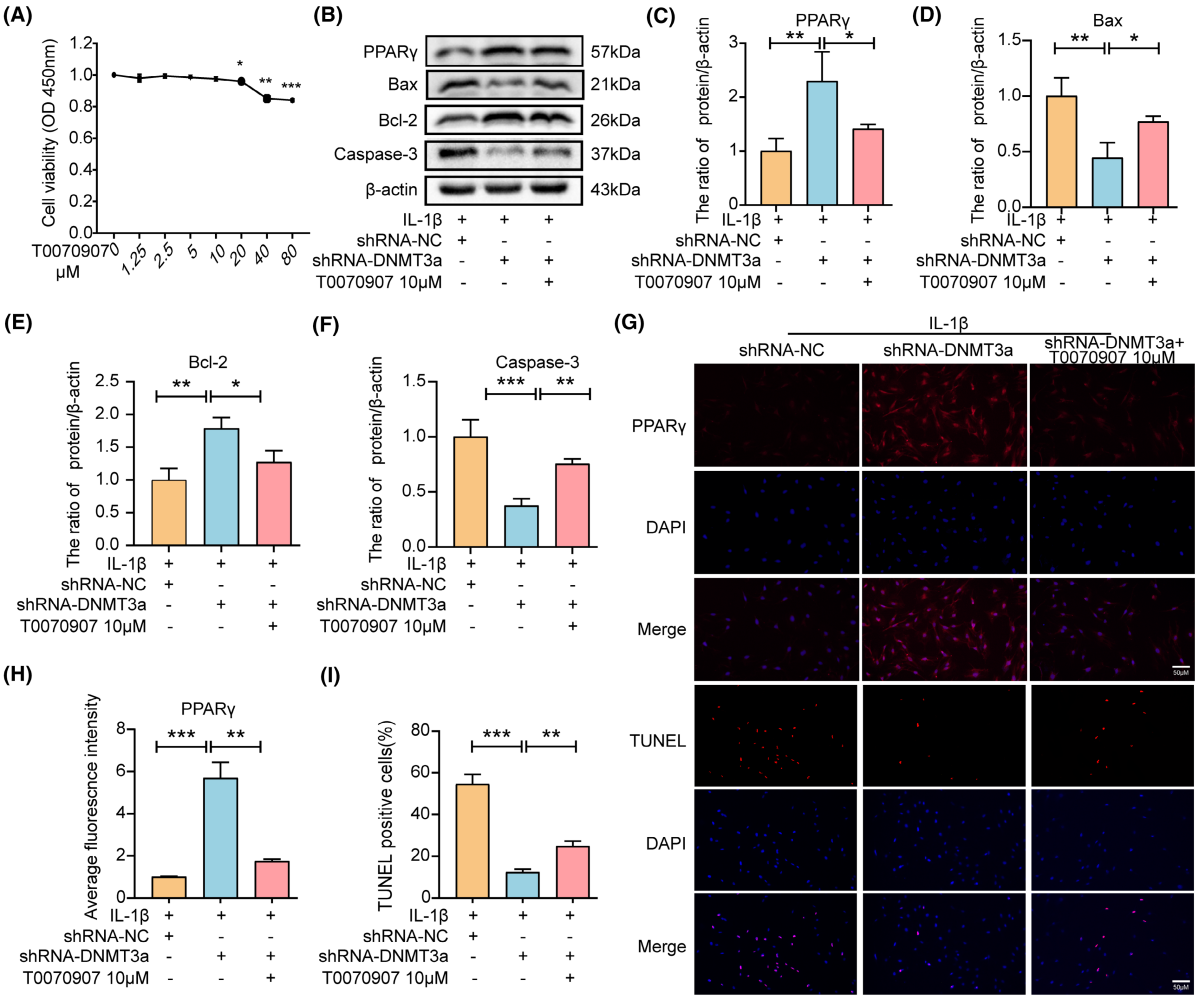
DNMT3a inhibits PPARγ to promote apoptosis in NP cells. (A) CCK‐8 was used to determine the cytotoxic effect of different concentrations of T0070907 on NP cells for 24 h; (B) Western blotting and quantitative analyses show changes in PPARγ, Bax, Bcl‐2, and Caspase‐3 in rat NP cells after T0070907 pretreatment; (C–F) Western blotting quantification of PPARγ, Bax, Bcl‐2, and Caspase‐3; (G, H) immunofluorescence and quantitative analyses show changes in PPARγ; (G, I) TUNEL staining and apoptotic cell ratio quantification. Three biological replicates were contained in each experiment. All data are expressed as mean ± standard deviation (SD); *p < 0.05, **p < 0.01, ***p < 0.01.

To determine whether DNMT3a exerts anti‐apoptotic effects via PPARγ, we pretreated NP cells with a specific inhibitor of PPARγ (T0070907). First, we detected the toxic effects of T0070907 on rat NP cells, and the results showed that no toxicity existed in NP cells below 10 μM (Figure 5A). Western blotting and immunofluorescence results showed that T0070907 inhibited PPARγ expression (Figure 5B,C,G,H). In addition, compared with the shRNA‐NC group, the shRNA‐DNMT3a group experienced significantly reduced IL‐1 β‐induced Bax and Caspase‐3 expression levels and increased PPARγ and Bcl‐2 expression levels. However, pretreatment with T0070907 reversed the increase in PPARγ owing to shRNA‐DNMT3a treatment and increased Bax and Caspase‐3 expression levels while reducing the expression of Bcl‐2, suggesting that inhibition of PPARγ expression eliminated the anti‐apoptotic effect of shRNA‐DNMT3a (*p* < 0.05) (Figure 5B,D–F). Similarly, the TUNEL staining results showed that DNMT3a inhibition significantly reduced the apoptosis rate, whereas T0070907 significantly reversed the anti‐apoptotic effect of shRNA‐DNMT3a (Figure 5G,I). These results indicate that DNMT3a inhibition regulates NP apoptosis through PPARγ.

**FIGURE 6:**
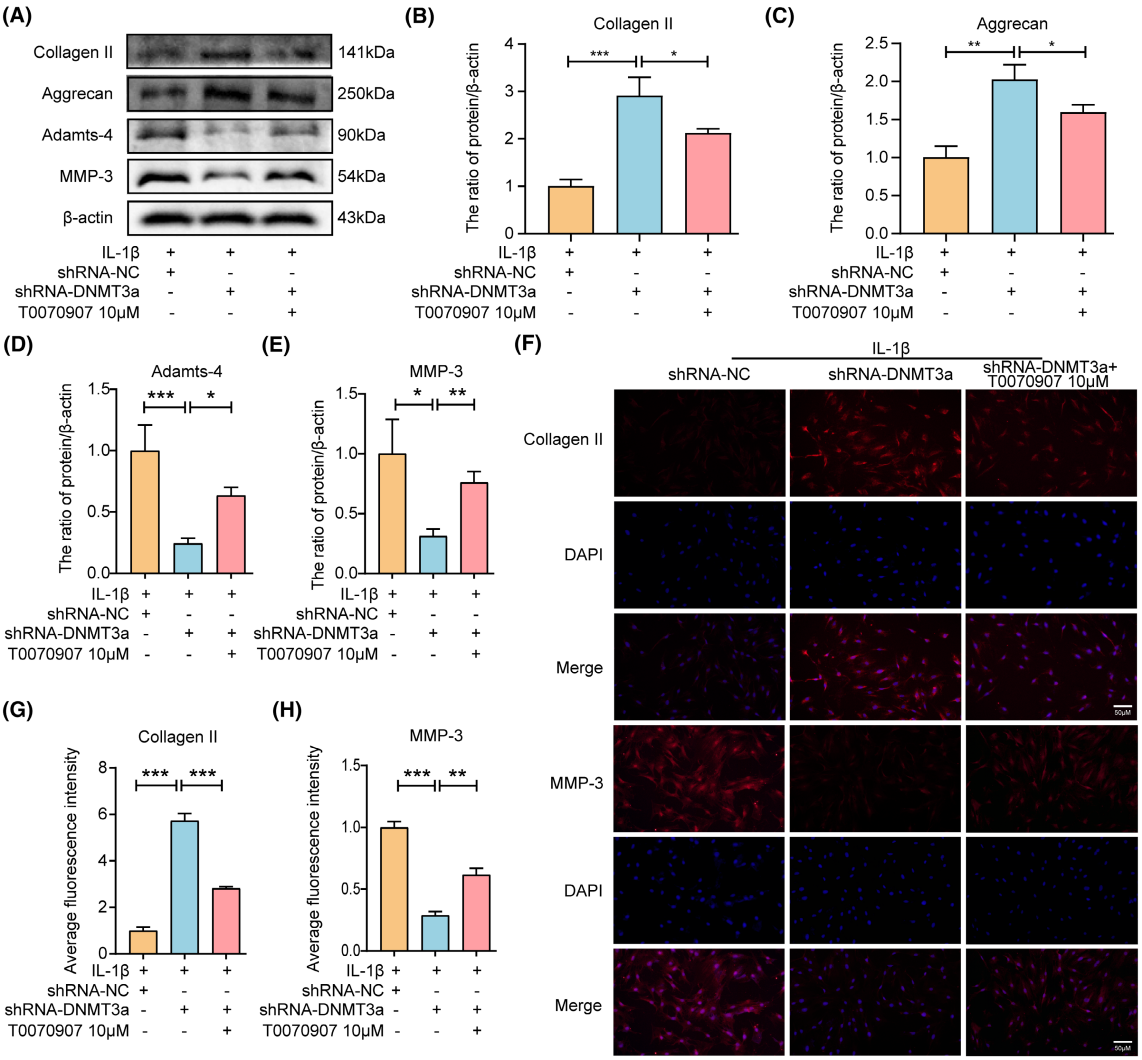
DNMT3a inhibits PPARγ to promote ECM degradation in NP cells. (A–E) Western blotting and quantitative analysis showed changes in Collagen II, Aggrecan, Adamts‐4, and MMP‐3 expression levels in rat NP cells after T0070907 pretreatment; (F–H) immunofluorescence and quantitative analysis showed changes in Collagen II and MMP‐3; Three biological replicates were contained in each experiment. All data are expressed as mean ± standard deviation (SD); *p < 0.05, **p < 0.01, ***p < 0.01.

In addition, to determine whether DNMT3a participates in anti‐ECM degradation via PPARγ, we pretreated NP cells by adding T0070907 to the medium. Western blotting showed that the shRNA‐DNMT3a group experienced significantly reduced IL‐1β‐induced Adamts‐4 and MMP‐3 expression levels and increased collagen II and aggrecan expression levels compared with the shRNA‐NC group. However, T0070907 eliminated the anti‐ECM degradation of shRNA‐DNMT3a by inhibiting PPARγ expression (Figure 6A–E). Similarly, immunofluorescence results showed that DNMT3a inhibition significantly increased collagen II and decreased MMP‐3 expression. The inhibitory effect of shRNA‐DNMT3a was reversed in the presence of T0070907 (Figure 6F–H). Thus, DNMT3a inhibition regulates the degradation of ECM in NP cells via PPARγ.

### 3.6 DNMT3a regulates the activity of the PPARγ/NF‐κB axis

**FIGURE 7:**
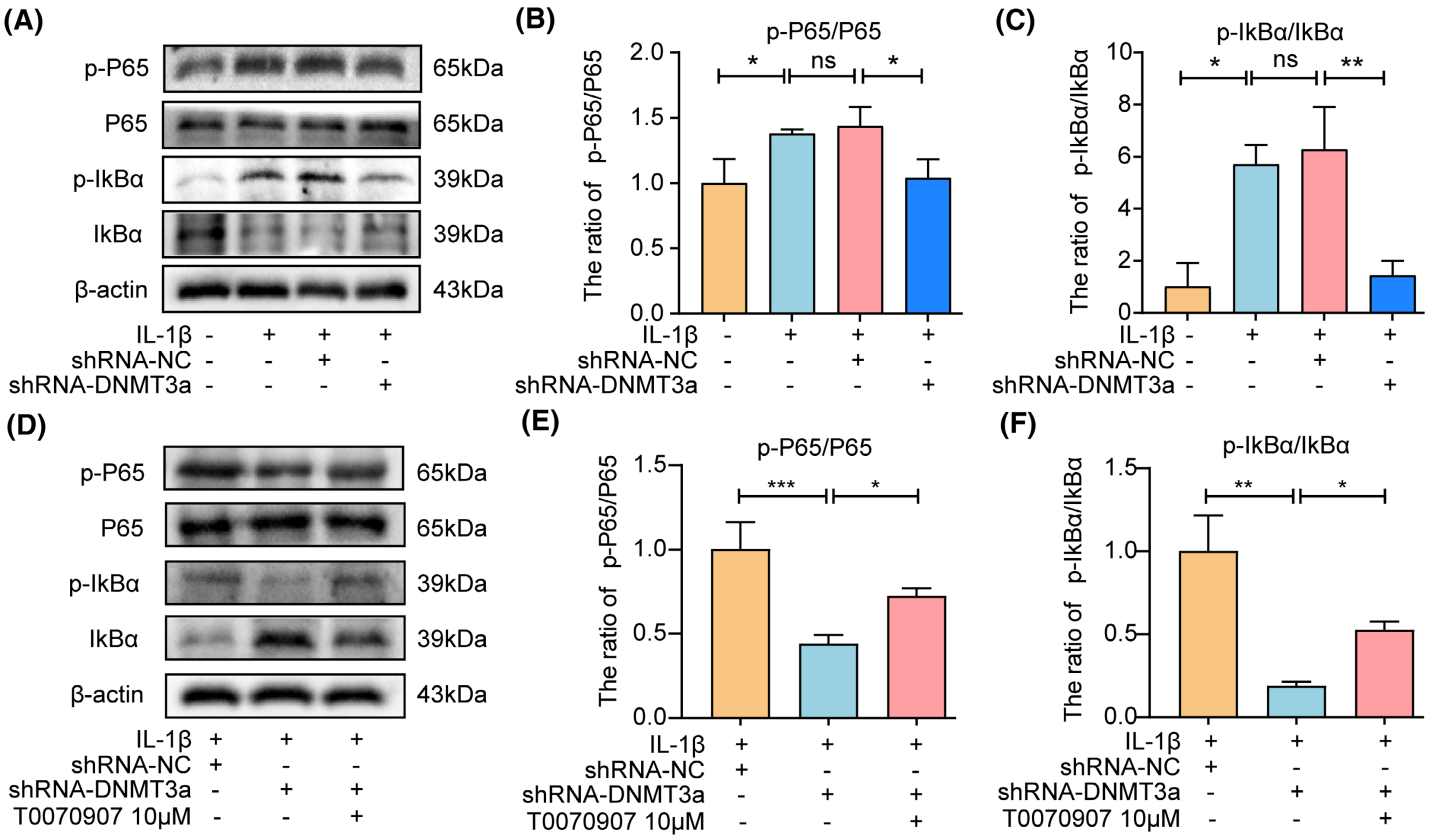
DNMT3a regulates the activity of the PPARγ/NF‐κB axis. (A–C) Western blotting and quantitative analyses show levels of p‐P65, P65, p‐IkBα, and IkBα in rat NP cells following IL‐1β and shRNA‐DNMT3a treatment; (D–F) Western blotting and quantitative analyses showed p‐P65, P65, p‐IkBα, and IkBα levels in rat NP cells after T0070907 pretreatment; Three biological replicates were contained in each experiment. All data are expressed as mean ± standard deviation (SD); *p < 0.05, **p < 0.01, ***p < 0.01, ns *p* > 0.05.

NF‐κB pathway plays a crucial role in the pathological processes of IVDD. PPARγ can regulate the activity of the NF‐κB pathway and participate in the ECM degradation of chondrocytes, anabolism, apoptosis and inflammation, and other pathological processes in osteoarthritis. However, whether PPARγ can participate in IVDD development by regulating the NF‐κB pathway activity remains unclear. To further explore the underlying molecular mechanism of PPARγ regulating apoptosis and ECM degradation in NP cells, we observed that DNMT3a inhibition significantly inhibited the activation of the NF‐κB pathway induced by IL‐1β compared with the IL‐1β and shRNA‐NC groups (Figure 7A–C), indicating that the NF‐κB pathway is involved in apoptosis and ECM degradation of NP cells. In addition, when T0070907 was pretreated, the inhibitory effect of DNMT3a inhibition on the NF‐κB pathway declined compared to that of shRNA‐DNMT3a (Figure 7D–F), indicating that DNMT3a regulates the activity of the PPARγ/NF‐κB axis.

### 3.7 PPARγ regulates apoptosis and ECM degradation by inhibiting the NF‐κB pathway

**FIGURE 8:**
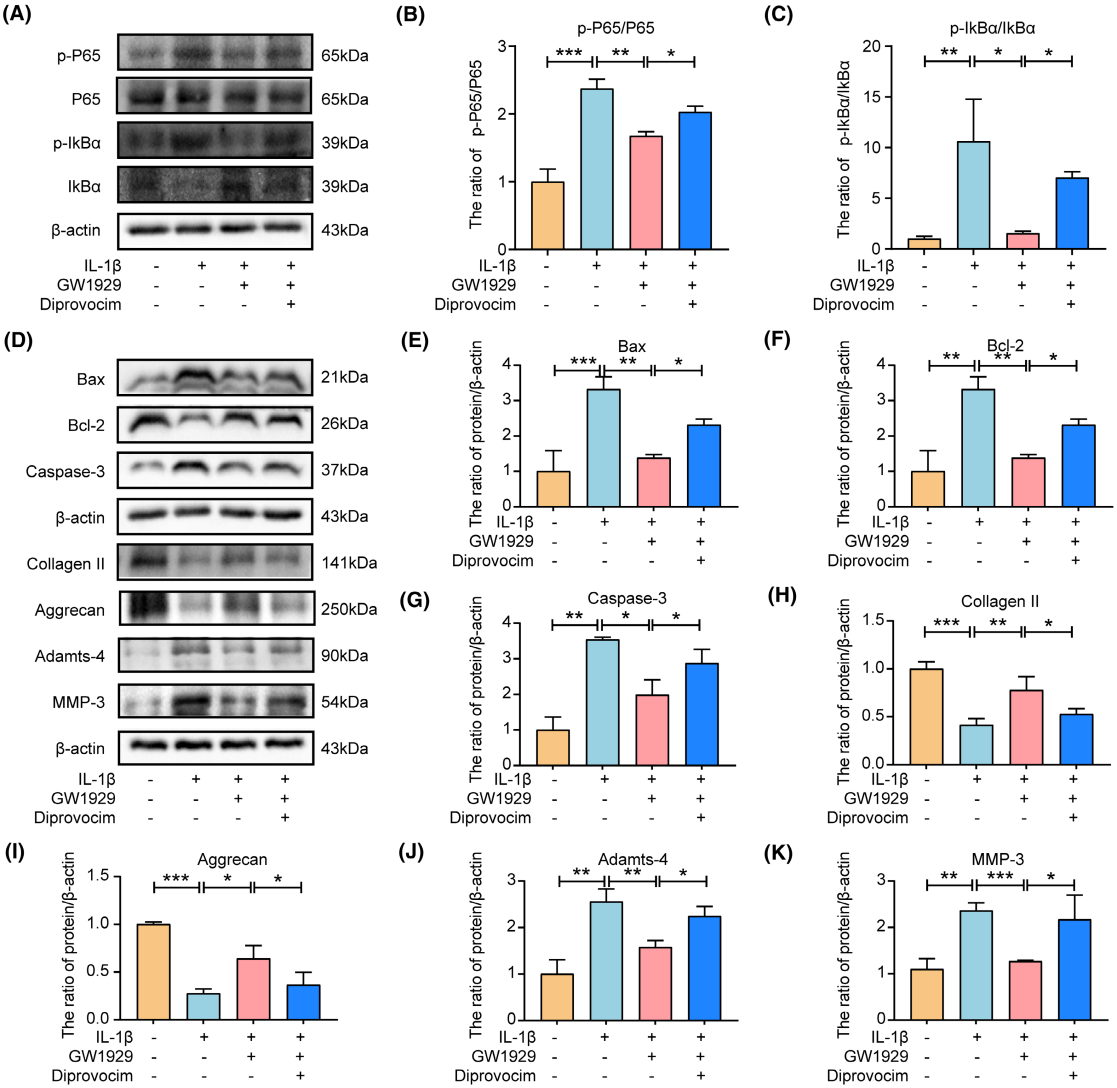
PPARγ regulates apoptosis and ECM degradation by inhibiting the NF‐κB pathway. (A–C) Western blotting and quantitative analysis showed p‐P65, P65, p‐IkBα, and IkBα levels in rat NP cells after treatment with GW1929 and Diprovocim; (D–K) western blotting and quantitative analyses show Bax, Bcl‐2, Caspase‐3, Collagen II, Aggrecan, Adamts‐4 and MMP‐3 levels treated with GW1929 and Diprovocim. Three biological replicates were contained in each experiment. All data are expressed as mean ± standard deviation (SD); *p < 0.05, **p < 0.01, ***p < 0.01.

We further verified whether PPARγ regulates apoptosis and ECM degradation in NP cells via the NF‐κB pathway. First, we examined the effects of PPARγ activator (GW1929) and NF‐κB pathway activator (diprovocim) on NP cell viability and observed that 10 μM GW1929 and 20 μM diprovocim were not toxic to NP cells (Supporting Information S2). Therefore, we chose GW1929 at a concentration of 10 μM and diprovocim at 20 μM for the relevant experimental studies. Figure 8A–C show that GW1929 pretreatment inhibits‐induced activation of the NF‐κB pathway induced by IL‐1β. In addition, western blotting showed that GW1929 significantly reduced the Bax and Caspase‐3 expression levels and increased the Bcl‐2 expression levels compared to IL‐1β group, while the anti‐apoptotic effect of GW1929 was debilitated by activating the NF‐κB pathway, indicating that PPARγ regulates the apoptosis of NP cells through the NF‐κB pathway (Figure 8D–K). Similarly, compared with the IL‐1β, GW1929 significantly reduced the Adamts‐4 and MMP‐3 expression levels and increased Collagen II and Aggrecan expression levels, while the anti‐ECM degradation of GW1929 was weakened by activating the NF‐κB pathway using diprovocim (Figure 8D–K). These results suggest that PPARγ regulated ECM degradation in NP cells through the NF‐κB pathway.

### 3.8 DNMT3a inhibition ameliorates puncture‐induced IVDD in vivo

**FIGURE 9:**
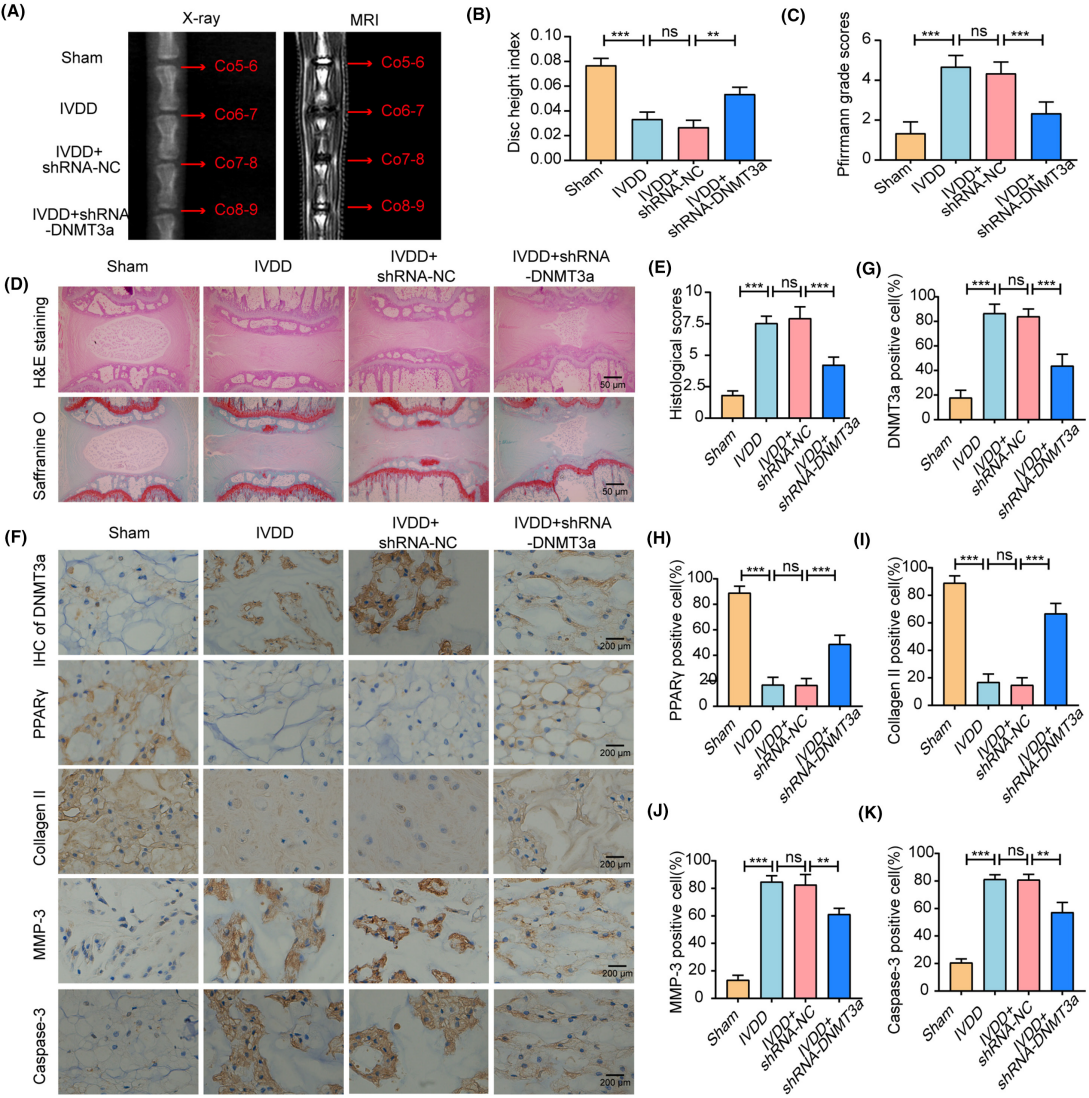
DNMT3a inhibition ameliorated puncture‐induced IVDD in vivo. (A) Representative X‐ray image and MRI T2 image of the intervertebral disc at 8 weeks after shRNA‐DNMT3a injection in rats; (B) the variation of the disc height index is calculated based on X‐ray images; (C) Pfirrmann grade scores calculated from MRI T2 images; (D, E) HE, Safranine O staining images and histological scores; (F–K) IHC staining and quantitative analysis of DNMT3a, PPARγ, Collagen II, MMP‐3 and Caspase‐3 in intervertebral disc samples from different groups of rats. Three biological replicates were contained in each experiment. All data are expressed as mean ± standard deviation (SD); **p < 0.01, ***p < 0.01 and ns *p* > 0.05.

To detect the protective effects of DNMT3a inhibition in rat intervertebral discs, we used a classical acupuncture‐induced IVDD model. The degree of IVDD in the rats was assessed using radiography, MRI and Pfirrmann grading. We mapped the stages of male Co5–6, Co6–7, Co7‐8, and Co8‐9 from each SD rat to the Sham, IVDD, IVDD+shRNA‐NC and IVDD+shRNA‐DNMT3a groups. Radiographic and MRI T2 images of the rat coccygeal vertebrae were acquired 8 weeks after surgery, and disc height measurements and Pfirrmann grades were performed (Figure 9A–C). The HE staining and saffranine O staining were performed on NP tissues of rats in different groups, and histological scores were performed (Figure 9D,E). This indicated that DNMT3a inhibition significantly improved the development of IVDD. In addition, further immunohistochemical analysis showed that the level of DNMT3a in the IVDD+shRNA‐DNMT3a group was significantly lower than that in the IVDD and IVDD+shRNA‐NC groups, indicating that shRNA‐DNMT3a could significantly reduce the expression of DNMT3a in the NP tissues of IVDD rats. More importantly, compared with the IVDD and IVDD+shRNA‐NC groups, the expressions of PPARγ and collagen II increased, while the expression levels of MMP‐3 and Caspase‐3 decreased in the IVDD+shRNA‐DNMT3a group (Figure 9F–K). This indicated that inhibition of DNMT3a could significantly inhibit ECM degradation and apoptosis. In summary, DNMT3a inhibition can increase the anabolism of ECM and decrease the apoptosis of NP cells, thus improving the ameliorate puncture‐induced IVDD in vivo.

## 4 DISCUSSION

LBP caused by IVDD is increasingly becoming a focus of medical attention.^31^ According to a statistical analysis of the 2019 Global Burden of Disease Study, LBP rose from 13th to 9th place between 1990 and 2019, and disability caused by LBP increased by 46.9%.^32^ Apoptosis and ECM degradation are crucial contributing factors in IVDD development.^33^, ^34^ Currently, it is difficult to fundamentally delay or reverse the further development of IVDD by clinical methods (conservative or surgical) treatment. Therefore, it is crucial to explore the potential pathogenic mechanisms underlying IVDD. Recent studies have revealed that multiple differential methylation sites in IVDD are associated with human disc degeneration^14^ and that DNMT3a modifications regulate various musculoskeletal disorders.^35^, ^36^ In this study, we first revealed that DNMT3a levels increased and PPARγ levels decreased gradually during the IVDD progression, suggesting an underlying regulatory relationship. In vitro experiments demonstrated that DNMT3a decreased PPARγ expression levels, which was caused by DNMT3a‐mediated promoter PPARγ hypermethylation. DNMT3a inhibition increased PPARγ expression and inhibited apoptosis and ECM degradation of IL‐1β‐induced NP cells. Further studies showed that PPARγ regulates apoptosis and ECM degradation by inhibiting the NF‐κB pathway in NP cells. Therefore, our results revealed that PPARγ inhibition was associated with abnormal methylation and may serve as a key target for IVDD treatment.

The role of DNMT3a‐mediated methylation in many diseases and pathological processes has been extensively studied. Li et al.^37^ found that DNMT3a can mediate TCF21/hnRNPA1 methylation by regulating the NF‐κB signalling pathway to aggravate liver fibrosis, suggesting that DNMT3a‐mediated methylation may play a critical role in the pathophysiology of the disease. PPARγ is involved in various physiopathological processes, which are closely related to the maintenance of homeostasis of chondrocytes. Interestingly, inhibition of DNA methylase levels (DNMT1, DNMT3a, and DNMT3b) promotes PPARγ expression and improves pulmonary fibrosis.^38^ Similarly, 5aza, a DNA methylase inhibitor, was found to attenuate the methylation of the PPARγ promoter region, thereby promoting PPARγ expression, maintaining homeostasis in chondrocytes, and delaying the osteoarthritis progression.^19^ Here, we described for the first time that the key DNA methylation regulator, DNMT3a, can affect the PPARγ expression by regulating CpG island methylation in the promoter region, revealing the underlying mechanism of epigenetic regulation of IVDD.

Apoptosis and ECM degradation are thought to be important factors that accelerate IVDD progression. A previous study found that the apoptosis rate in degenerated NP tissues was significantly higher than that in the early IVDD group and that excessive apoptosis affected the homeostasis of the IVD tissue structure.^39^, ^40^ Increased ECM degradation is often accompanied by increased levels of Adamts and MMPs‐degrading enzymes.^41^ Therefore, under the action of adverse factors, an increase in Adamts and MMPs degradation enzymes in NP cells leads to increased degradation of collagen II and aggrecan, thereby accelerating the occurrence and development of IVDD.^42^ Therefore, the inhibition of apoptosis and ECM degradation may be an attractive therapeutic strategy for IVDD. In this study, our silencing of DNMT3a significantly inhibited apoptosis and ECM degradation in IL‐1β‐induced NP cells. In vitro experiments further confirmed that the protective effect of DNMT3a against apoptosis and ECM degradation diminished in the presence of an inhibitor of PPARγ (T0070907), indicating that DNMT3a regulates apoptosis and ECM degradation in IL‐1β‐induced NP cells by PPARγ.

However, the specific downstream regulatory mechanism of DNMT3a‐mediated PPARγ methylation in regulating apoptosis and ECM degradation in NP cells remains unclear and deserves further in‐depth study. The NF‐κB pathway is considered an important signalling pathway within cells primarily because it can regulate various pathophysiological processes such as cell inflammation, apoptosis, ECM degradation and aging.^43^, ^44^, ^45^ Zheng et al.^46^ reported that S100A8/A9 binds to TLR4 to increase the expression of MMPs, TNF‐α, and IL‐6 in NP cells through the NF‐κB signalling pathway, delaying the IVDD progression. Similarly, Zou et al.^47^ found that HO‐1 expression was lower in the NP cells of patients with IVDD than in that of those in the early IVDD group. Additionally, in vitro experiments further revealed that the underlying mechanism of HO‐1 delay in IVDD inhibits IL‐1β‐induced apoptosis by promoting the formation of Beclin‐1/PI3KC3 complexes to promote autophagy and inhibit NF‐κB activity. Consistent with previous studies, we treated NP cells with GW1929 and diprovocim to investigate the regulatory relationship between the PPARγ and NF‐κB pathways. Our results showed that PPARγ inhibition can accelerate IVDD development by activating the NF‐κB signalling pathway by phosphorylating p65. However, we demonstrated that upregulation of PPARγ inhibited the activation of NF‐κB signalling and attenuated IL‐1β‐induced apoptosis and ECM degradation and that the protective effect of PPARγ diminished in the presence of NF‐κB pathway activators. Conclusively, these findings describe for the first time that DNMT3a in NP cells can mediate the PPARγ/NF‐κB axis activity, participating in NP apoptosis and anabolic catabolism of ECM.

This study had some limitations. First, grades I and II were mainly obtained from patients with idiopathic scoliosis and lumbar fractures, and the sample size of early human NP tissues was relatively small. Second, genetic factors and disc sampling may vary. Finally, in the in vitro experiments, we selected normal rat NP cells for functional experimental verification; however, there were certain differences between species, and further studies are needed in the future.

Summarily, this study revealed that PPARγ has a protective effect against apoptosis and ECM degradation of NP. In vitro, DNMT3a inhibits demethylation of the PPARγ promoter region and the activation of the NF‐κB signalling pathway. Thereby reducing apoptosis and ECM degradation in NP cells. In vivo, DNMT3a inhibition also had a protective effect against IVDD. We believe that the DNMT3a mediated‐PPARγ/NF‐κB axis may provide new insights into the potential pathogenesis of IVDD and may be an attractive target for the treatment of IVDD.

## AUTHOR CONTRIBUTIONS

**Peng Cheng:** Data curation (lead); software (lead); writing – original draft (lead); writing – review and editing (lead). **Hang‐Zhi Wei:** Data curation (supporting); investigation (supporting). **Hai‐Wei Chen:** Data curation (supporting); software (supporting). **Zhi‐Qiang Wang:** Data curation (supporting); software (supporting). **Peng Mao:** Investigation (supporting); methodology (supporting); visualization (supporting). **Hai‐Hong Zhang:** Conceptualization (equal); supervision (equal); visualization (equal).

## FUNDING INFORMATION

This work was supported by the Natural science foundation of Gansu Province (22JR11RA061), the Gansu Province Key research and Development program–Social development category (23YFFA0042).

## CONFLICT OF INTEREST STATEMENT

The authors declare that there are no conficts of interest.

## Supporting information


Data S1.

## Data Availability

The data used to support the findings of this study are available from the corresponding author upon request.

## References

[1] Guo Q , Zhu D , Wang Y , et al. Targeting STING attenuates ROS induced intervertebral disc degeneration. Osteoarthr Cartil. 2021;29(8):1213‐1224. 10.1016/j.joca.2021.04.017 34020031

[2] Xing H , Zhang Z , Mao Q , et al. Injectable exosome‐functionalized extracellular matrix hydrogel for metabolism balance and pyroptosis regulation in intervertebral disc degeneration. J Nanobiotechnology. 2021;19(1):264. 34488795 10.1186/s12951-021-00991-5 PMC8419940

[3] Binch ALA , Fitzgerald JC , Growney EA , Barry F . Cell‐based strategies for IVD repair: clinical progress and translational obstacles. Nat Rev Rheumatol. 2021;17(3):158‐175. 33526926 10.1038/s41584-020-00568-w

[4] Roh EJ , Darai A , Kyung JW , et al. Genetic therapy for intervertebral disc degeneration. Int J Mol Sci. 2021;22(4):1579. 33557287 10.3390/ijms22041579 PMC7914740

[5] Hu ZL , Li HY , Chang X , et al. Exosomes derived from stem cells as an emerging therapeutic strategy for intervertebral disc degeneration. World J Stem Cells. 2020;12(8):803‐813. 32952860 10.4252/wjsc.v12.i8.803 PMC7477652

[6] Feng C , Liu H , Yang M , Zhang Y , Huang B , Zhou Y . Disc cell senescence in intervertebral disc degeneration: causes and molecular pathways. Cell Cycle. 2016;15(13):1674‐1684. 27192096 10.1080/15384101.2016.1152433 PMC4957599

[7] Guo S , Cui L , Xiao C , et al. The mechanisms and functions of GDF‐5 in intervertebral disc degeneration. Orthop Surg. 2021;13(3):734‐741. 33817978 10.1111/os.12942 PMC8126946

[8] Li G , Zhang W , Liang H , Yang C . Epigenetic regulation in intervertebral disc degeneration. Trends Mol Med. 2022;28(10):803‐805. 36030154 10.1016/j.molmed.2022.07.007

[9] Kang L , Zhang H , Jia C , Zhang R , Shen C . Epigenetic modifications of inflammation in intervertebral disc degeneration. Ageing Res Rev. 2023;87:101902. 36871778 10.1016/j.arr.2023.101902

[10] Bird A . Perceptions of epigenetics. Nature. 2007;447(7143):396‐398. 17522671 10.1038/nature05913

[11] Vidaurre V , Chen X . Epigenetic regulation of drosophila germline stem cell maintenance and differentiation. Dev Biol. 2021;473:105‐118. 33610541 10.1016/j.ydbio.2021.02.003 PMC7992187

[12] Reik W , Dean W , Walter J . Epigenetic reprogramming in mammalian development. Science. 2001;293(5532):1089‐1093. 11498579 10.1126/science.1063443

[13] Wu H , Zhang Y . Reversing DNA methylation: mechanisms, genomics, and biological functions. Cell. 2014;156(1–2):45‐68. 24439369 10.1016/j.cell.2013.12.019 PMC3938284

[14] Ikuno A , Akeda K , Takebayashi SI , Shimaoka M , Okumura K , Sudo A . Genome‐wide analysis of DNA methylation profile identifies differentially methylated loci associated with human intervertebral disc degeneration. PLoS One. 2019;14(9):e0222188. 31513634 10.1371/journal.pone.0222188 PMC6742346

[15] Li G , Luo R , Zhang W , et al. m6A hypomethylation of DNMT3B regulated by ALKBH5 promotes intervertebral disc degeneration via E4F1 deficiency. Clin Transl Med. 2022;12(3):e765. 35340126 10.1002/ctm2.765 PMC8957938

[16] Wu L , Guo C , Wu J . Therapeutic potential of PPARγ natural agonists in liver diseases. J Cell Mol Med. 2020;24(5):2736‐2748. 32031298 10.1111/jcmm.15028 PMC7077554

[17] Zaiou M . Peroxisome proliferator‐activated receptor‐γ as a target and regulator of epigenetic mechanisms in nonalcoholic fatty liver disease. Cell. 2023;12(8):1205. 10.3390/cells12081205 PMC10136748 37190114

[18] Vasheghani F , Zhang Y , Li YH , et al. PPARγ deficiency results in severe, accelerated osteoarthritis associated with aberrant mTOR signalling in the articular cartilage. Ann Rheum Dis. 2015;74(3):569‐578. 25573665 10.1136/annrheumdis-2014-205743 PMC4345902

[19] Zhu X , Chen F , Lu K , Wei A , Jiang Q , Cao W . PPARγ preservation via promoter demethylation alleviates osteoarthritis in mice. Ann Rheum Dis. 2019;78(10):1420‐1429. 31239244 10.1136/annrheumdis-2018-214940

[20] Liu Y , Qu Y , Liu L , et al. PPAR‐γ agonist pioglitazone protects against IL‐17 induced intervertebral disc inflammation and degeneration via suppression of NF‐κB signaling pathway. Int Immunopharmacol. 2019;72:138‐147. 30981079 10.1016/j.intimp.2019.04.012

[21] Zhang G , Wang H , Zhang Q , Zhao Z , Zhu W , Zuo X . Bergenin alleviates H(2) O(2) ‐induced oxidative stress and apoptosis in nucleus pulposus cells: involvement of the PPAR‐γ/NF‐κB pathway. Environ Toxicol. 2021;36(12):2541‐2550. 34499403 10.1002/tox.23368

[22] Zhang GZ , Liu MQ , Chen HW , et al. NF‐κB signalling pathways in nucleus pulposus cell function and intervertebral disc degeneration. Cell Prolif. 2021;54(7):e13057. 34028920 10.1111/cpr.13057 PMC8249791

[23] Chen W , Deng Z , Zhu J , et al. Rosuvastatin suppresses TNF‐α‐induced matrix catabolism, pyroptosis and senescence via the HMGB1/NF‐κB signaling pathway in nucleus pulposus cells. Acta Biochim Biophys Sin (Shanghai). 2023;55(5):795‐808. 37222533 10.3724/abbs.2023026 PMC10281883

[24] Li B , Yang X , Zhang P , et al. Engeletin alleviates the inflammation and apoptosis in intervertebral disc degeneration via inhibiting the NF‐κB and MAPK pathways. J Inflamm Res. 2022;15:5767‐5783. 36238766 10.2147/JIR.S371809 PMC9553281

[25] Urrutia J , Besa P , Campos M , et al. The Pfirrmann classification of lumbar intervertebral disc degeneration: an independent inter‐ and intra‐observer agreement assessment. Eur Spine J. 2016;25(9):2728‐2733. 26879918 10.1007/s00586-016-4438-z

[26] Chen HW , Liu MQ , Zhang GZ , et al. Proanthocyanidins inhibit the apoptosis and aging of nucleus pulposus cells through the PI3K/Akt pathway delaying intervertebral disc degeneration. Connect Tissue Res. 2022;63(6):650‐662. 35491814 10.1080/03008207.2022.2063121

[27] Lin J , du J , Wu X , et al. SIRT3 mitigates intervertebral disc degeneration by delaying oxidative stress‐induced senescence of nucleus pulposus cells. J Cell Physiol. 2021;236(9):6441‐6456. 33565085 10.1002/jcp.30319

[28] Li H , Wang X , Pan H , et al. The mechanisms and functions of IL‐1β in intervertebral disc degeneration. Exp Gerontol. 2023;177:112181. 37088216 10.1016/j.exger.2023.112181

[29] Krupkova O , Hlavna M , Amir Tahmasseb J , et al. An inflammatory nucleus pulposus tissue culture model to test molecular regenerative therapies: validation with epigallocatechin 3‐gallate. Int J Mol Sci. 2016;17(10):1640. 27689996 10.3390/ijms17101640 PMC5085673

[30] Johnson ZI , Schoepflin ZR , Choi H , Shapiro IM . Disc in flames: roles of TNF‐α and IL‐1β in intervertebral disc degeneration. Eur Cell Mater. 2015;30:104‐116. discussion 116‐7. 26388614 10.22203/ecm.v030a08 PMC4751407

[31] Wei J , Ou Z , Tong B , Liao Z , Yang C . Engineered extracellular vesicles as therapeutics of degenerative orthopedic diseases. Front Bioeng Biotechnol. 2023;11:1162263. 37362216 10.3389/fbioe.2023.1162263 PMC10289007

[32] Global burden of 369 diseases and injuries in 204 countries and territories, 1990–2019: a systematic analysis for the global burden of disease study 2019. Lancet. 2020;396(10258):1204‐1222. 33069326 10.1016/S0140-6736(20)30925-9 PMC7567026

[33] Zhu G , Yang XW , Zhou WW , Lian X , Hao YJ . PLAGL2 induces nucleus pulposus cell apoptosis via regulating RASSF5 expression and thus accelerates intervertebral disc degeneration. Exp Cell Res. 2023;430(1):113699. 37364764 10.1016/j.yexcr.2023.113699

[34] Dou X , Ma Y , Luo Q , et al. Therapeutic potential of melatonin in the intervertebral disc degeneration through inhibiting the ferroptosis of nucleus pulpous cells. J Cell Mol Med. 2023;27:2340‐2353. 37329158 10.1111/jcmm.17818 PMC10424295

[35] Zhang Z , Yang B , Zhou S , Wu J . CircRNA circ_SEC24A upregulates DNMT3A expression by sponging miR‐26b‐5p to aggravate osteoarthritis progression. Int Immunopharmacol. 2021;99:107957. 34325283 10.1016/j.intimp.2021.107957

[36] Gaur N , Karouzakis E , Glück S , et al. MicroRNAs interfere with DNA methylation in rheumatoid arthritis synovial fibroblasts. RMD Open. 2016;2(2):e000299. 27843576 10.1136/rmdopen-2016-000299 PMC5073550

[37] Li L , Diao S , Chen Z , et al. DNMT3a‐mediated methylation of TCF21/hnRNPA1 aggravates hepatic fibrosis by regulating the NF‐κB signaling pathway. Pharmacol Res. 2023;193:106808. 37268177 10.1016/j.phrs.2023.106808

[38] Wei A , Gao Q , Chen F , et al. Inhibition of DNA methylation de‐represses peroxisome proliferator‐activated receptor‐γ and attenuates pulmonary fibrosis. Br J Pharmacol. 2022;179(7):1304‐1318. 34378791 10.1111/bph.15655

[39] Zhang XB , Hu YC , Cheng P , et al. Targeted therapy for intervertebral disc degeneration: inhibiting apoptosis is a promising treatment strategy. Int J Med Sci. 2021;18(13):2799‐2813. 34220308 10.7150/ijms.59171 PMC8241771

[40] Li H , Tian L , Li J , et al. The roles of circRNAs in intervertebral disc degeneration: inflammation, extracellular matrix metabolism, and apoptosis. Anal Cell Pathol (Amst). 2022;2022:9550499. 35186669 10.1155/2022/9550499 PMC8856834

[41] Wang WJ , Yu XH , Wang C , et al. MMPs and ADAMTSs in intervertebral disc degeneration. Clin Chim Acta. 2015;448:238‐246. 26162271 10.1016/j.cca.2015.06.023

[42] Liang H , Luo R , Li G , Zhang W , Song Y , Yang C . The proteolysis of ECM in intervertebral disc degeneration. Int J Mol Sci. 2022;23(3):1715. 35163637 10.3390/ijms23031715 PMC8835917

[43] Lawrence T . The nuclear factor NF‐kappaB pathway in inflammation. Cold Spring Harb Perspect Biol. 2009;1(6):a001651. 20457564 10.1101/cshperspect.a001651 PMC2882124

[44] Cao Y , Tang S' , Nie X , et al. Decreased miR‐214‐3p activates NF‐κB pathway and aggravates osteoarthritis progression. EBioMedicine. 2021;65:103283. 33714889 10.1016/j.ebiom.2021.103283 PMC7957119

[45] Sivandzade F , Prasad S , Bhalerao A , Cucullo L . NRF2 and NF‐қB interplay in cerebrovascular and neurodegenerative disorders: molecular mechanisms and possible therapeutic approaches. Redox Biol. 2019;21:101059. 10.1016/j.redox.2018.11.017 PMC6302038 30576920

[46] Zheng J , Wang J , Liu H , et al. Alarmins S100A8/A9 promote intervertebral disc degeneration and inflammation‐related pain in a rat model through toll‐like receptor‐4 and activation of the NF‐κB signaling pathway. Osteoarthr Cartil. 2022;30(7):998‐1011. 10.1016/j.joca.2022.03.011 35405347

[47] Zou L , Lei H , Shen J , et al. HO‐1 induced autophagy protects against IL‐1 β‐mediated apoptosis in human nucleus pulposus cells by inhibiting NF‐κB. Aging (Albany NY). 2020;12(3):2440‐2452. 32015215 10.18632/aging.102753 PMC7041769

