# Supplementary material for: DNMT3a‐mediated methylation of PPARγ promote intervertebral disc degeneration by regulating the NF‐κB pathway
Source: J Cell Mol Med. 2023 Nov 20;28(2):e18048. doi: 10.1111/jcmm.18048 (PMC10826446; doi:10.1111/jcmm.18048)
Supplement: Supplementary file 1 — Data S1. [file JCMM-28-e18048-s001.docx]

**
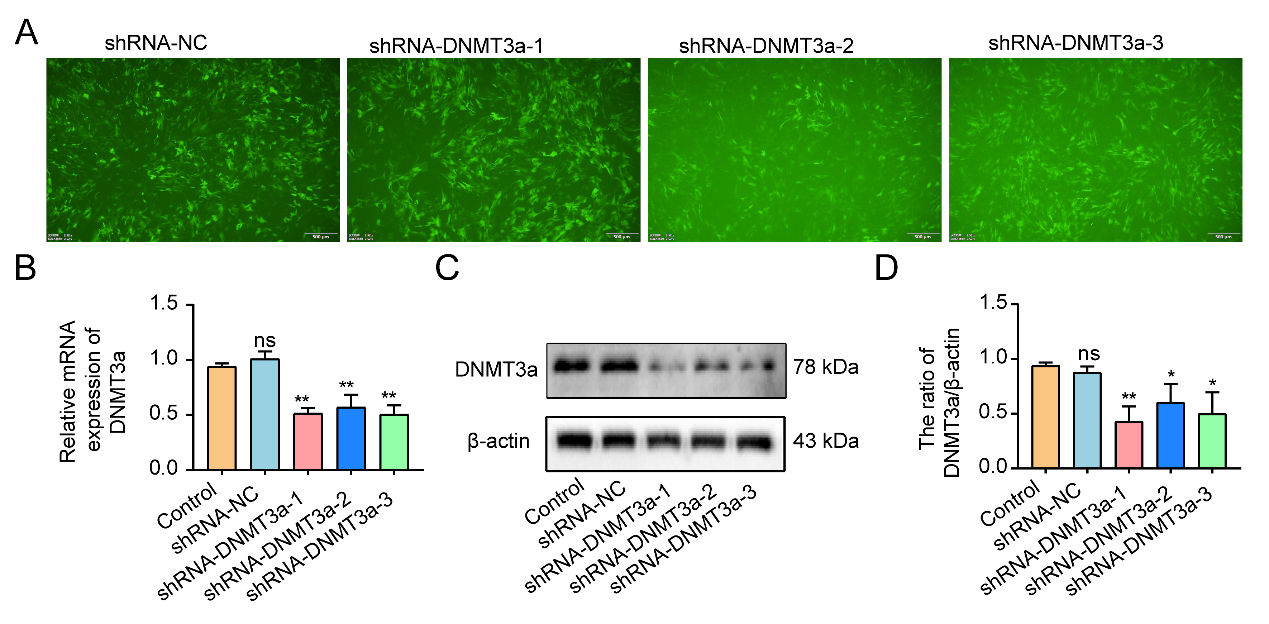
**

**Supplementary material.1** Detection of transfection efficiency of DNMT3a. . (A) Rat NPCs transfected with lentivirus successfully showed green fluorescence under the GFP channel of the fluorescence microscope. (B) RT-qPCR was used to evaluate the mRNA expression of DNMT3a in different groups after transfection; (C-D), western blotting was used to evaluate the expression of DNMT3a in different groups after transfection.


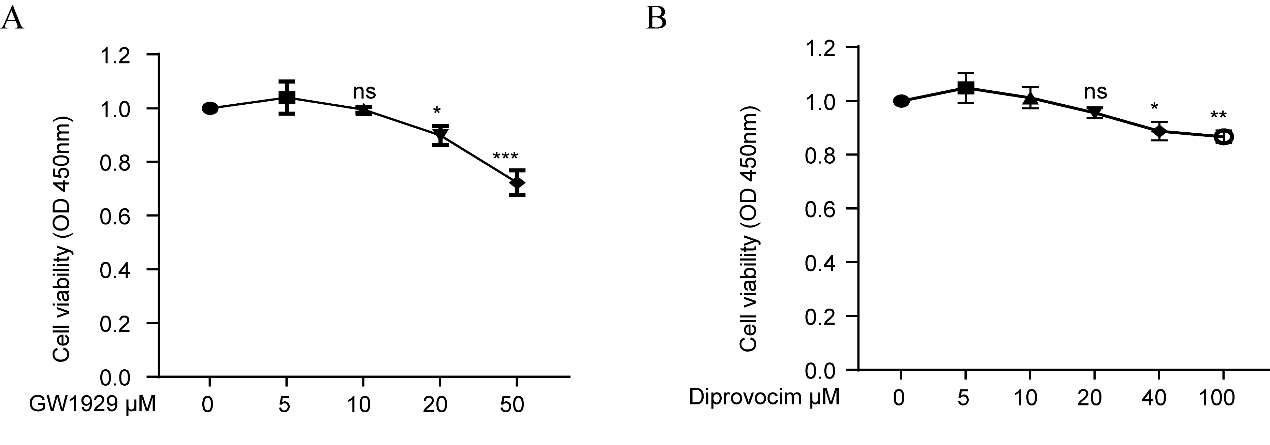


**Supplementary material.2** Cell viability of GW1929 and Diprovocim. (A), Cell viability of of GW1929 on rat NP cells; (B), Cell viability of of Diprovocim on rat NP cells;
